# Exosomes as a potential therapeutic approach in osteoimmunology

**DOI:** 10.3389/fimmu.2023.1309015

**Published:** 2023-12-19

**Authors:** Ivan V. Zhivodernikov, Yuliya V. Markina, Tatiana V. Kirichenko, Mikhail A. Popov, Alexander M. Markin

**Affiliations:** ^1^Laboratory of Cellular and Molecular Pathology of Cardiovascular System, Petrovsky National Research Center of Surgery, Moscow, Russia; ^2^Department of Cardiac Surgery, Moscow Regional Research and Clinical Institute (MONIKI), Moscow, Russia; ^3^Medical Institute, Poples’ Friendship University of Russia named after Patrice Lumumba (RUDN University), Moscow, Russia

**Keywords:** exosomes, osteoimmunology, target therapy, immune system, osteoporosis

## Abstract

Exosomes are natural extracellular vesicles that play a key role in inter- and intracellular communication. Currently they are considered as a promising therapeutic strategy for the treatment of various diseases. In osteoimmunology, exosomes can serve as biomarkers of bone homeostasis disorders and, at the same time, promising therapeutic agents with high stability in the biological environment, low immunogenicity and good bioavailability. In this review, we attempted to examine exosomes as natural mediators of intercellular communication, playing an essential role in the interaction of the immune system and bone tissue, based on an analysis of the PubMed database up to October 2023.

## 1 Introduction

Osteoimmunology is a field of research that studies the interaction between immune system cells and bone tissue, including various types of cells and associated signaling pathways. The term was introduced by Aaron and Choi in 2000 (1). Immune cells play a key role in the development, formation, and resorption of bone tissue, thereby regulating bone homeostasis. Consequently, imbalance in the interaction of immune cells can lead to the development of bone diseases (2). Osteoporosis is one of the most common systemic diseases characterized by bone mass loss due to bone remodeling performed by osteoblasts, osteoclasts, osteocytes, and their precursors. In turn, extracellular vesicles, including exosomes, play a significant role in the development of osteoporosis. Exosomes are crucial for inter- and intracellular communication and act as regulatory molecules and potential biomarkers for bone mass loss (3, 4). The role of exosomes in osteogenesis with the involvement of exosomal microRNAs and proteins was demonstrated in several studies. In an LPS-induced model of bone mass loss in mice, an increase in circulating exosomes and a change in exosomal microRNA expression were observed, which significantly inhibited osteogenic differentiation (5). In this review, we attempted to examine exosomes as natural mediators of intercellular communication, playing an essential role in the interaction of the immune system and bone tissue, based on an analysis of the PubMed database up to October 2023.

## 2 The role of the immune system in the pathogenesis of bone diseases

### 2.1 Cellular mechanism of bone remodeling

During growth and development, the mineralized bone matrix is continuously destroying and new intercellular substance is being synthesized by osteoblasts, followed by osteocyte differentiation and mineralization. Continuous bone remodeling allows bones growing, leads to damage repair and better adaptation the bone structure to physical loads by orienting the bone trabeculae according to the load vector. Trabecular alignment according to physical loads leads to anisotropy of bone, increasing strength in the direction of these loads. This remodeling results in a significant increase in strength without increasing bone mass, thus increasing the efficiency of the bone structure (6). Remodeling is accomplished by two mechanisms, bone resorption and formation of new bone tissue in place of resorbed matrix. Osteoclasts are macrophage-like cells responsible for the resorption of bone tissue that induces bone remodeling. Normally, there is a balance between osteoblasts proliferation with production of an extracellular matrix for bone formation and bone resorption by osteoclasts (7, 8). Osteoclasts move through the bone tissue, making “tunnels”, breaking down the mineralized matrix with the help of hydrolytic enzymes - acid phosphatase and tartrate-dependent acid phosphatase. Following osteoclasts, osteoblasts move in bone tunnels and produce extracellular matrix. Osteoblasts and osteoclasts are interconnected and their joint movement through bone is called bone remodeling unit (BMU). The proliferation and maturation of osteoclasts are mainly regulated by receptor activator of nuclear factor-kappaB (NF-κB) ligand (RANKL) from the TNF family (9).

### 2.2 Estrogen deficiency reveals the interconnectedness of the bone and immune systems

**Figure 1:**
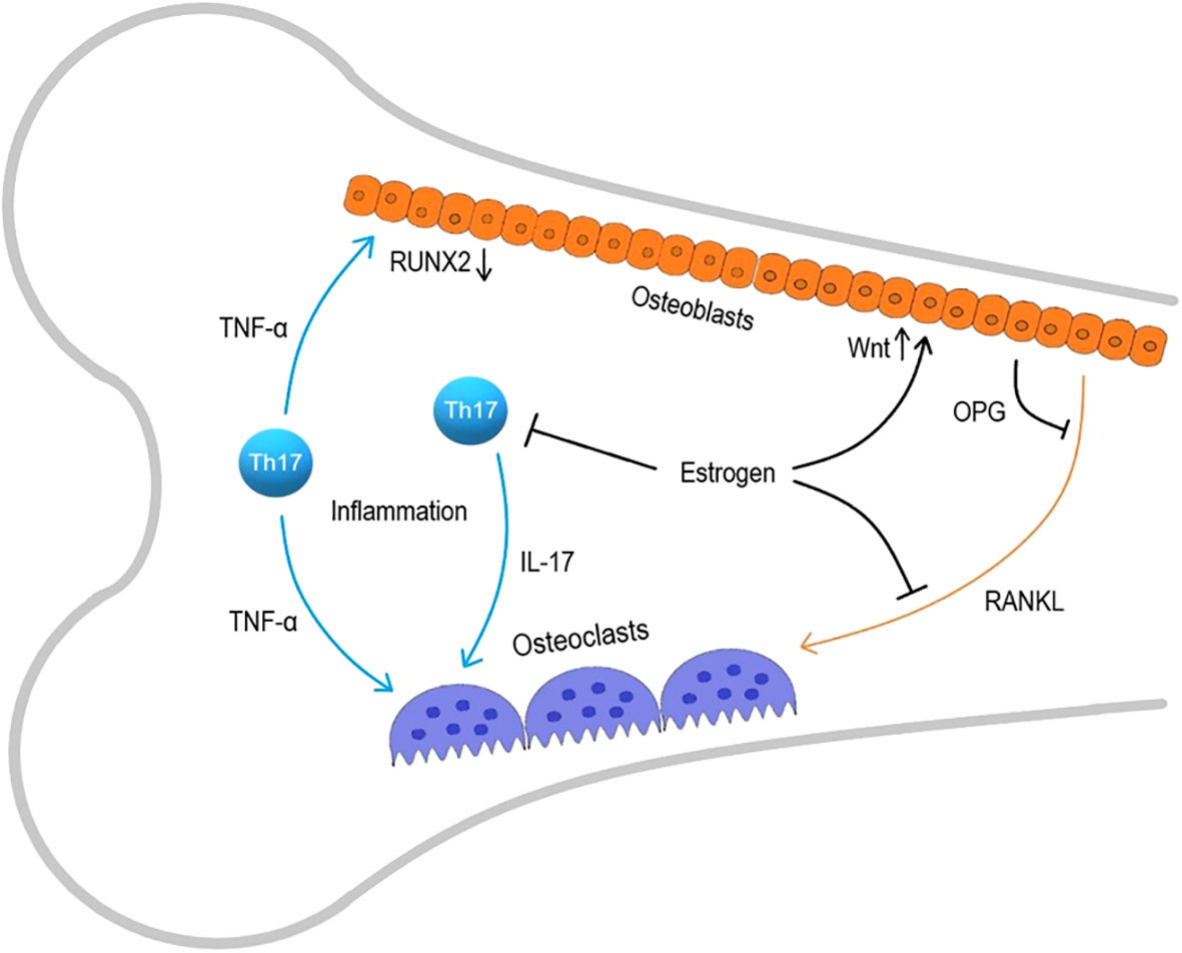
Immune mechanisms in osteoporosis development. RANKL, receptor activator of nuclear factor kappa-B ligand; Th-17, T helper 17.

Inflammation is characterized by the activation of immune cells and an increased production of pro-inflammatory cytokines. In acute inflammation, there is a short-term increase in the secretion of inflammatory mediators by activated immune cells, but in chronic inflammation, the persistent activation of immune cells is observed. In chronic inflammatory conditions, the number of circulating monocytes and T-cells increases along with elevated concentrations of interleukins IL-1, IL-6, IL-8, INF-γ, tumor necrosis factor- α (TNF-α), and other pro-inflammatory mediators, which activate immune cells. During chronic inflammation, elevated TNF-α levels induce osteoclast proliferation and activation, although RANKL is the major mediator for osteoclasts (9). The effects of factors of the TNF family, to which RANKL belongs, may partially overlap due to similar binding domains; moreover, macrophages activated by TNF-α and osteoclasts activated by RANKL are differentiated from monocytes. Therefore, osteoclasts are sensitive to TNF-α, whereas RANKL is involved in maintaining dendritic cell survival and immune system functioning (10, 11). Prolonged changes in the cytokine profile alter the functioning of many cells outside the immune system, especially osteoclasts (12). Effector T-cells, especially Th-17 cells produce IL-17 that play a crucial role in osteoclast activation. Experiments on thymus-less mice with a T-cell deficit have shown that T-cell deficiency provides the protection from bone mass loss and increased bone tissue metabolism caused by ovariectomy, highlighting the key role of the T-cell component of osteoimmunity (13). Osteoclast activation and disruption of bone homeostasis are especially pronounced during estrogen deficiency in postmenopause, that stimulates the development of osteoporosis through activation of immune system. The immune mechanisms in the development of osteoporosis are presented at **Figure 1**.

Among multiple effects in organism, estrogens limit the lifespan of T-cells and the antigen-independent activation of memory T-cells producing TNFα and IL-17A (14). Estrogens also suppress RANKL production and increase the production of osteoprotegerin (OPG), a RANKL antagonist, by osteoblasts and lymphocytes, maintaining the RANKL/OPG ratio essential for balanced bone remodeling (15–17). It’s also shown that estrogen sustains the vitality of early osteoblasts by enhancing the Wnt signaling pathway, while TNF-α increased in estrogen deficiency conditions, inhibits osteoblast differentiation by reducing RUNX2 expression (18, 19). Prolonged inflammation adversely affects maintaining bone tissue structure, especially against the backdrop of estrogen deficiency, that is why the postmenopausal state is so closely linked to osteoporosis.

### 2.3 Proinflammatory activation of the immune system in aging

The second pro-inflammatory “blow” of the immune system on bone tissue occurs due to changes in the ratio of immunocompetent cells. With age, the adaptive component of immunity weakens and the number of T- and B-lymphocytes decreases, while the innate component of immune system increases, leading to chronic inflammation. Weakening of the adaptive component of the immune system results in decreased resistance to new infections and response to vaccines. Thymus involution occurs partly due to loss of antigen-dependent priming of T-lymphocytes by dendritic cells; in addition, lymphocyte production in the bone marrow decreases due to a reduced pool of hematopoietic stem cells (20). Despite the decrease in the number of B-lymphocytes, the level of immunoglobulins circulating in the blood increases, but these antibodies are characterized by lower affinity, high polyspecificity and autologous reactivity. As a result, autoimmune diseases associated with systemic inflammation such as rheumatoid arthritis, vasculitis, and systemic lupus erythematosus are more common in the elderly and correlate with osteoporosis as a consequence of increased production of proinflammatory mediators such as IL-1, IL-6, CRP, and TNF-α (21). IL-17 production also increases due to a shift in CD4+ T cell differentiation to Th17 instead of Th1 and Th2, and IL-17 is known to be able to activate osteoclasts. To attempt to reverse the aging of thymus and increase the pool of T cells, antioxidants and blockers of cyclin-dependent kinases are of considerable interest, somatotropin, IGF-1, IL-7 and FGF7 have also been studied in this regard (22).

## 3 Exosomes in osteoimmunology

### 3.1 Characteristics of exosomes

Exosomes are natural membrane-bound nano-sized structures secreted by almost all living cells and transport various biologically active molecules to recipient cells. The size of exosomes ranges from 30-150 nm, in contrast to apoptotic bodies (500-2000 nm) released from the plasma membrane during apoptosis, and microvesicles (100-1000 nm), which are also secreted by cells (23, 24). Exosomes, like cells, are covered with a lipid bilayer and are released into the extracellular space after the fusion of multivesicular bodies with plasma membranes (25). Exosomes are secreted by many types of cells, including hematopoietic cells (B cells, T cells, dendritic cells, mast cells, platelets), glandular cells, immune, endothelial, and mesenchymal stem cells (MSC), among others (26). Exosomes can be detected in almost all types of body fluids, such as blood, saliva, urine, synovial fluid, pleural effusions of ascites, bronchoalveolar lavage fluid, amniotic fluid, and breast milk (27). The main known natural functions of exosomal vesicles within the body are the removal of unnecessary cellular materials and facilitating intercellular communication through the transfer of macromolecules (28). The content of exosomes depends on the type of cell, as well as on signaling regulatory mechanisms. Various proteins, such as receptors, enzymes, transcription factors, as well as nucleic acids (mRNA, miRNA) and lipids can be found both on the membrane and inside the exosomes. Some of these components are common to different types of exosomes, while others depend on the source tissue (29).

Endocytosis leads to the creation of early endosomes, which capture cellular proteins and genetic materials found in the cytoplasm, then transform into late endosomes, from which multivesicular bodies are generated (30). Multivesicular bodies then either break down with lysosomes or fuse with the plasma membrane, releasing nanovesicles into the extracellular space (31). Exosomes can carry antibodies and other membrane-bound signaling molecules on their surface. After secretion from parent cells, exosomes can initiate downstream signaling cascades through receptor-ligand interaction with cell surface proteins or release their contents through endocytosis after merging with the plasma membrane of recipient cells, thus facilitating the transfer of intercellular signals. Some types of extracellular vesicles are capable of transporting organelles, such as mitochondria and ribosomes (32, 33).

### 3.2 Potential for therapeutic applications of exosomes

The therapeutic application of exosomes is of interest to many scientists since exosomes can stably exist in various body fluids, carrying specific biomolecules and protecting them from degradation (34, 35). Exosomes can be used as biomarkers in diagnostic of various diseases, as drug delivery vehicles or therapeutic agents, and as immunomodulators that stimulate or suppress the immune system (36). The use of exosomes as natural carriers for delivering drugs or nucleic acids to target cells is considered as a promising direction in the development of treatment approaches in various diseases. Exosomes can express specific surface proteins or be loaded with a therapeutic agent, ensuring targeted delivery to affected tissues and minimizing off-target effects (37, 38). The structure of exosomes allows encapsulating hydrophilic active therapeutic agents in the core and hydrophobic compounds in the lipid bilayer, though this remains one of the significant challenges in their development (39). Moreover, the use of exosomes as therapeutic agents avoids the major limitations of cell therapy (40). Advantages of exosomes also include their stability, low immunogenicity, and biocompatibility (41). The absence of the risk of immune rejection and malignancy, stability, long-term maintenance, and the ability to overcome biological barriers are distinctive features characterizing exosomes (42). These unique properties of exosomes provide new therapeutic strategies for treating various diseases. It’s well-known that drug delivery across the blood-brain barrier is challenging as it’s impermeable to most therapeutic agents. It has been proven that cell-derived exosomes or bioengineered exosomes loaded with drugs can penetrate the blood-brain barrier and can be stable in peripheral circulation (43). Moreover, exosomes can cross the maternal-placental barrier, making them an attractive treatment medium for various diseases (44).

### 3.3 Exosome effects on bone remodeling

**Figure 2:**
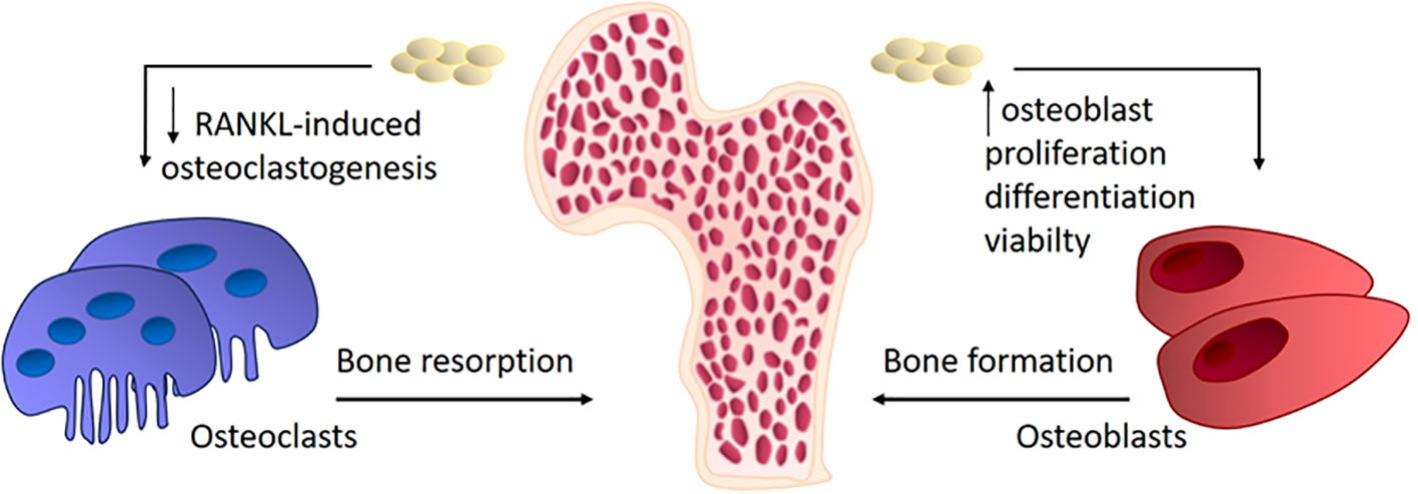
The mechanisms of exosomes effects on bone remodeling in osteoporosis.

As previously mentioned, the most common bone tissue disease is osteoporosis. The fundamental factor in the pathogenesis of osteoporosis is the aberrant differentiation of osteoclasts (45). Osteoporosis is a metabolic bone disease characterized by a deterioration of bone tissue structure, a reduction in bone mass, and a predisposition to fractures (46). Exosomes have great potential for clinical use due to their immunocompatibility (47). The therapeutic potential of exosomes obtained from osteoblast cultures is currently of great interest. It was shown that adding exosomes, derived from other osteoblasts and stem cells, to an osteoblast culture enhances their differentiation and viability. The beneficial effects of application of exosomes on bone morphology were demonstrated in experimental animals. The positive effect of using osteoblasts, MSCs, and exosomes has been demonstrated in the treatment of induced osteoporosis in rabbits. However, this study had some limitations, such as the non-equivalent number of exosomes compared to cells, which did not allow to evaluate objectively the therapeutic effect of exosomes (48). The composition and functionality of exosomes depending on their cellular origin has been demonstrated in some studies through their effects on rat bone density and osteoblast differentiation. Exosomes from umbilical cord blood stem cells and exosomes from the same cells in an osteodifferentiated state had different effects on the differentiation of rat skull osteoblast cultures. Exosomes obtained from native cells stimulated osteoblast proliferation, while exosomes obtained from cells in osteogenic conditions promoted osteoblast differentiation to a greater extent. However, in mice with osteoporosis induced by ovariectomy, both types of exosomes improved the density of the tibial bone and reversed osteoporosis *in vivo* (49). Studies demonstrating the efficacy of exosomes’ action on the mechanisms underlying osteoporosis and osteopenia are associated with the detection of active microRNA or its inhibitor. For example, microRNA-133 can inhibit the differentiation of MSCs into osteoblasts by reducing the expression of Runx2 (50). lncRNA MALAT1 (metastasis-associated lung adenocarcinoma transcript 1) stimulates the expression of osterix and the osteogenesis of MSCs by absorbing miRNA-143 (51). Runx2 is the primary transcription factor responsible for the osteogenic differentiation of precursors, functioning through the transcription factor osterix. Donor MSC exosomes transferred microRNA-151-5p to the bone marrow of recipient mice, activating the osteogenic differentiation of bone marrow MSCs and thus alleviating osteopenia in mice. The same effect is achieved by systemically introduced miR-151-5p (52). In addition to influencing the viability, proliferation and differentiation of osteoblasts, exosomes may be effective on the opposite side of bone tissue homeostasis, namely, osteoclastogenesis. The potential mechanisms of exosome effects on bone remodeling are presented at **Figure 2**. Exosomal miRNA-214-3p from osteoclasts inhibits osteogenesis, and inhibiting miRNA-214-3p with antisense microRNA reverses the effect (53). Overall, exosomes appear to be an attractive tool for the safe and effective delivery of RNA-based drugs. In addition to protecting RNA from the aggressive internal environment of the body, they reduce toxicity and immunogenicity and also demonstrate high stability in blood plasma (54).

It was shown in some studies that exosomes derived from MSCs subjected to cyclic stretching inhibited RANKL-induced osteoclastogenesis of macrophages by suppressing the NF-κB signaling pathway (55). Exosomes derived from tumor cells have been shown to activate osteoclastogenesis, explaining some mechanisms of osteoporosis onset in oncology (56–59). Research data indicates that microRNAs such as miR-935, miR-21-5p, miR-27a-5p, miR-31a-5p, miR-29a, and miR-146a, which are encapsulated in exosomes *in vivo*, participate in bone tissue remodeling (60). Exosome-encapsulated miR-21a-5p was used in the design of an experimental bone implant and contributed to its osteointegration, as well as macrophage polarization towards the anti-inflammatory M2 phenotype, enhancing osteogenesis in a rat femoral bone drilling model (61). Macrophage polarization was observed in a similar experimental implant model but without microRNA detection. Exosomes from bone marrow MSCs, introduced into the polyetheretherketone implant possessed the osteoimmunomodulatory effect (62). Thus, exosome functionality can be explained in the context of bone and immune system interaction. The addition of exosomes from T-cells of patients with osteoporosis to osteoblasts, reduced the expression of genes associated with osteogenic differentiation - RUNX2, COL1, OC, OP (63). In mice, in a model of bone mass loss induced by lipopolysaccharides, an increase in the number of exosomes loaded with miRNA-125b-5p, miRNA-132-3p, and miRNA-214-3p inhibited the osteogenesis of progenitor cells was observed (5). Exosomal regulation was identified in the functioning of myeloid suppressors, cells responsible for macrophage polarization from M1 to M2 and weakening Th1 and Th17 immune responses, which is important for the suppression of osteoclastogenesis and bone resorption. In the presence of exosomes from myeloid immunosuppressors, CD8+ T cells demonstrate anergy, moreover, they have found significant accompanying factors of osteogenesis - TGF-β and IL-10 (64).

According to some research, exosomes from immune cells (e.g., dendritic cells, T cells, B cells, and natural killers) can be considered as promising immunomodulators. Today, the immunomodulatory properties of exosomes are of great interest as a potential therapy for bone diseases. It was shown in some studies that MSC-derived exosomes possess a strong effect on the immune system through increased microRNA expression, modulation of angiogenesis, and anti-inflammatory action (65, 66). In addition, exosomes have an immunomodulatory secretome which is capable to reduce the proliferation of synoviocytes and macrophages, as well as to produce the pro-inflammatory cytokines upon *in vitro* stimulation. In a model of acute inflammation of the synovial membrane/IFP (the infrapatellar fat pad) in rats, therapeutic treatment with exosomes from MSCs led to stable polarization of macrophages towards an anti-inflammatory therapeutic M2 phenotype in the tissues of the synovial membrane/IFP (66).

In the context of age-related changes, chronic inflammation accompanied by insufficient repolarization of M1 macrophages to M2 leads to increased activation of osteoclasts and decreased formation of osteoblasts, thereby increasing bone resorption. MSC-derived exosomes are able to modulate the immune response and polarize macrophages to an anti-inflammatory M2 phenotype. Exosomes contain cytokines of various origins that are involved in bone repair processes and also stimulate the expression of osteoblast differentiation genes, accelerating the resolution of inflammation (67). The modulatory effect of exosomes is to alter macrophage polarization by influencing nuclear factor-κB (NF-κB), a key transcription factor in macrophages (68). In addition, exosomes isolated in monocytes transport arachidonic acid, a precursor of the inflammatory factor PGE2, which enhances the secretion of IL-10 and stimulates the polarization of M2 macrophages, thereby exerting an anti-inflammatory effect (69). It is worth noting that exposure to NF-κB can also have a negative effect since its activation leads to the expression of inflammatory cytokines IL-1β and TNF-α, which can aggravate the inflammatory process in bone (70). Thus, exosomes play an important role in the regulation of inflammatory bone diseases, but the full spectrum of inflammatory cytokines associated with exosomes is not fully known.

In addition to immunomodulating properties through the influence of various cytokines, exosomes can inhibit the processes of cell apoptosis. Studies have shown that exosomes secreted by bone marrow-derived mesenchymal stromal cells (BMSCs) attenuated TNF-α-induced cytotoxicity and apoptosis in cells MC3T3-E1 osteoblast is the most commonly used *in vitro* model of bone matrix mineralization (71). Other studies have also shown that BMSC exosomes lead to attenuation of osteoporosis by reducing the expression of miR-150-3p, which promotes osteoblast proliferation and differentiation, and inhibits apoptosis (72).

## 4 Limitations and future prospects for the use of exosomes

The role of exosomes in the body has been repeatedly confirmed experimentally, but active research is being conducted to clarify their specific functions in certain physiological and pathological conditions. Unlike traditional therapy for bone diseases, exosome therapy has several advantages. In particular, this is the absence or low immunogenicity of these molecules. In addition, it was shown in several studies that exosomes derived from MSCs promote bone formation, and their use is accompanied by the absence of side effects and low toxicity (73). It is worth noting that exosomes have good bioavailability and the potential to target specific cells and tissues. For example, osteoclast exosomes are able to target osteoblasts using one of the bone cell interaction mediators EphrinA2 (74).

However, despite the promising prospects for using exosomes as therapeutic agents, there are a number of limitations and challenges. To date, there are no clinically approved therapies using exosomes (73). Since exosomes carry a group of signaling molecules, the administration of exosomes may trigger various immune responses (75, 76). In addition, another important parameter is the sample from which exosomes are obtained, which determines their regulatory potential. In this regard, the main challenge currently is to evaluate the immunogenicity, increase the bioavailability and target exosomes to specific tissues and organs, in particular bone tissue (76, 77). In general, exosomes are representative of the state of the cells from which they are synthesized and play a crucial role in stabilizing this state. Exosomes derived from MSCs and other stem cells are of interest because they often exhibit anti-inflammatory effects by polarizing macrophages towards the M2 phenotype, increasing the number of regulatory T cells, increasing cell viability and activating osteogenesis (78). In addition, exosomes containing microRNAs are promising. Of all the regulatory molecules, microRNA is the least stable in the internal environment of the body; the effectiveness of exosomes against a specific disease is determined by the content of specific microRNAs (79).

Summarizing the above, it’s worth noting that the prospects for the use of exosomes are not only in the treatment of bone diseases, but many other diseases, the key factor of which is inflammation. However, before clinical use of exosomes, it is necessary to select their stable characteristics, efficiency, precise targeting, safety, routes of administration, taking into account cost-effective approaches to their production.

## 5 Conclusion

Currently exosomes are considered not only as natural extracellular vesicles that play a key role in inter- and intracellular communication but also as a promising therapeutic strategy for the treatment of various diseases. The disruptions in the regulation of bone homeostasis by immune cells lead to the development of bone tissue diseases, including osteoporosis. Exosomes in osteoimmunology serve as biomarkers for bone mass loss and, at the same time, as promising therapeutic agents, possessing high stability in the biological environment, low immunogenicity, and good bioavailability. Experimental studies have demonstrated the therapeutic effects of exosomes, derived from various sources, on the functioning of bone tissue cells. On the one hand, the use of exosomes promotes proliferation, improved viability and differentiation of osteoblasts, providing a beneficial effect of exosomes on bone formation; on the other hand, exosomes are shown to be able to suppress the immune mechanisms of osteoclastogenesis, accompanied by bone resorption. However, for the direct clinical application of exosomes, many issues still need to be addressed. Standardization, the choice of extraction source, manipulating composition, specific characteristics, stability, biodistribution, and targeting of exosomes will contribute to enhancing their therapeutic potential and effective personalized therapy for various diseases.

## Author contributions

IZ: Visualization, Writing – original draft. YM: Writing – original draft, Writing – review & editing. TK: Visualization, Writing – review & editing. MP: Writing – review & editing. AM: Funding acquisition, Supervision, Writing – review & editing.

## References

[1] Ye Z Wang Y Xiang B Wang H Tao H Zhang C. Roles of the Siglec family in bone and bone homeostasis. Biomed Pharmacother (2023) 165:115064. doi: 10.1016/J.BIOPHA.2023.115064 37413904

[2] Ye K Zhang X Li S Liu X Nie X Qiao Y. Manganese-implanted titanium modulates the crosstalk between bone marrow mesenchymal stem cells and macrophages to improve osteogenesis. J Funct Biomater (2023) 14:456. doi: 10.3390/JFB14090456/S1 37754870 PMC10531852

[3] de Oliveira MC Heredia JE da Silva FRF Macari S. Extracellular vesicles in bone remodeling and osteoporosis. Adv Exp Med Biol (2023) 1418:155–68. doi: 10.1007/978-981-99-1443-2_11/FIGURES/2 37603279

[4] dos Anjos Pultz B Andrés Cordero da Luz F Socorro Peixoto Ferreira de Souza L Cristina Brígido Tavares P Alonso Goulart V. The multifaceted role of extracellular vesicles in metastasis: Priming the soil for seeding. Int J Cancer (2017) 140:2397–407. doi: 10.1002/IJC.30595 28090647

[5] Wang Y Zhang L Wang K Zhou H Li G Xu L. Circulating exosomes from mice with LPS-induced bone loss inhibit osteoblast differentiation. Calcif Tissue Int (2022) 111:185–95. doi: 10.1007/S00223-022-00977-X PMC9300544 35435443

[6] Palumbo C Ferretti M. *In vitro* and in vivo bone remodeling models: complementary pieces of the same puzzle. Stem Cell Rev Rep (2023) 19(8):2994–5. doi: 10.1007/S12015-023-10625-Y 37721658

[7] Li G Liu S Chen Y Xu H Qi T Xiong A. Teriparatide ameliorates articular cartilage degradation and aberrant subchondral bone remodeling in DMM mice. J Orthop Translat (2022) 38:241–55. doi: 10.1016/J.JOT.2022.10.015 PMC9731868 36514714

[8] Mudri D Bilić Ćurčić I Meštrović L Mihaljević I Kizivat T. Hyperthyroidism and Wnt signaling pathway: influence on bone remodeling. Metabolites (2023) 13(2):241. doi: 10.3390/METABO13020241 36837860 PMC9968154

[9] Wu D Cline-Smith A Shashkova E Perla A Katyal A Aurora R. T-cell mediated inflammation in postmenopausal osteoporosis. Front Immunol (2021) 12:687551. doi: 10.3389/FIMMU.2021.687551 34276675 PMC8278518

[10] Zhivodernikov IV Kirichenko TV Markina YV Postnov AY Markin AM. Molecular and cellular mechanisms of osteoporosis. Int J Mol Sci (2023) 24(21):15772. doi: 10.3390/IJMS242115772 37958752 PMC10648156

[11] Ta HM Nguyen GTT Jin HM Choi J Park H Kim N. Structure-based development of a receptor activator of nuclear factor-kappaB ligand (RANKL) inhibitor peptide and molecular basis for osteopetrosis. Proc Natl Acad Sci U S A (2010) 107:20281–6. doi: 10.1073/PNAS.1011686107 PMC2996688 21059944

[12] Zhang J Jiang J Qin Y Zhang Y Wu Y Xu H. Systemic immune-inflammation index is associated with decreased bone mass density and osteoporosis in postmenopausal women but not in premenopausal women. Endocr Connect (2023) 12(2):e220461. doi: 10.1530/EC-22-0461 36598289 PMC9986387

[13] Cline-Smith A Axelbaum A Shashkova E Chakraborty M Sanford J Panesar P. Ovariectomy activates chronic low-grade inflammation mediated by memory T cells, which promotes osteoporosis in mice. J Bone Miner Res (2020) 35:1174–87. doi: 10.1002/JBMR.3966 PMC8061311 31995253

[14] Fischer V Haffner-Luntzer M. Interaction between bone and immune cells: Implications for postmenopausal osteoporosis. Semin Cell Dev Biol (2022) 123:14–21. doi: 10.1016/J.SEMCDB.2021.05.014 34024716

[15] Tian H Jiang T Yang K Ning R Wang T Zhou Q. α-asarone attenuates osteoclastogenesis and prevents against estrogen-deficiency induced osteoporosis. Front Pharmacol (2022) 13:780590. doi: 10.3389/FPHAR.2022.780590 35370648 PMC8971932

[16] Wang LT Chen LR Chen KH. Hormone-related and drug-induced osteoporosis: A cellular and molecular overview. Int J Mol Sci (2023) 24(6):5814. doi: 10.3390/IJMS24065814 36982891 PMC10054048

[17] Cheng CH Chen LR Chen KH. Osteoporosis due to hormone imbalance: an overview of the effects of estrogen deficiency and glucocorticoid overuse on bone turnover. Int J Mol Sci (2022) 23(3):1376. doi: 10.3390/IJMS23031376 35163300 PMC8836058

[18] Gilbert L He X Farmer P Rubin J Drissi H Van Wijnen AJ. Expression of the osteoblast differentiation factor RUNX2 (Cbfa1/AML3/Pebp2alpha A) is inhibited by tumor necrosis factor-alpha. J Biol Chem (2002) 277:2695–701. doi: 10.1074/JBC.M106339200 11723115

[19] Ikedo A Imai Y. Estrogen receptor α in mature osteoblasts regulates the late stage of bone regeneration. Biochem Biophys Res Commun (2021) 559:238–44. doi: 10.1016/J.BBRC.2021.04.112 33964733

[20] Sauce D Larsen M Fastenackels S Duperrier A Keller M Grubeck-Loebenstein B. Evidence of premature immune aging in patients thymectomized during early childhood. J Clin Invest (2009) 119:3070–8. doi: 10.1172/JCI39269 PMC2752077 19770514

[21] Cabling MG Sandhu VK Downey CD Torralba KD. Cardiovascular disease and bone health in aging female rheumatic disease populations: A review. Womens Health (Lond) (2023) 19:17455057231155286. doi: 10.1177/17455057231155286 36825447 PMC9969471

[22] Montecino-Rodriguez E Berent-Maoz B Dorshkind K. Causes, consequences, and reversal of immune system aging. J Clin Invest (2013) 123:958–65. doi: 10.1172/JCI64096 PMC3582124 23454758

[23] Théry C Witwer KW Aikawa E Alcaraz MJ Anderson JD Andriantsitohaina R. Minimal information for studies of extracellular vesicles 2018 (MISEV2018): a position statement of the International Society for Extracellular Vesicles and update of the MISEV2014 guidelines. J Extracell Vesicles (2018) 7(1):1535750. doi: 10.1080/20013078.2018.1535750 30637094 PMC6322352

[24] Mathieu M Martin-Jaular L Lavieu G Théry C. Specificities of secretion and uptake of exosomes and other extracellular vesicles for cell-to-cell communication. Nat Cell Biol (2019) 21:9–17. doi: 10.1038/S41556-018-0250-9 30602770

[25] Hessvik NP Llorente A. Current knowledge on exosome biogenesis and release. Cell Mol Life Sci (2018) 75:193. doi: 10.1007/S00018-017-2595-9 28733901 PMC5756260

[26] Kalluri R LeBleu VS. The biology, function , and biomedical applications of exosomes. Science (2020) 367(6478):eaau6977. doi: 10.1126/SCIENCE.AAU6977 32029601 PMC7717626

[27] Vlassov AV Magdaleno S Setterquist R Conrad R. Exosomes: Current knowledge of their composition, biological functions, and diagnostic and therapeutic potentials. Biochim Biophys Acta Gen Subj (2012) 1820:940–8. doi: 10.1016/j.bbagen.2012.03.017 22503788

[28] Edgar JR. Q&A: What are exosomes, exactly? BMC Biol (2016) 14:46. doi: 10.1186/S12915-016-0268-Z 27296830 PMC4906597

[29] Mashouri L Yousefi H Aref AR Ahadi AM Molaei F Alahari SK. Exosomes: composition, biogenesis, and mechanisms in cancer metastasis and drug resistance. Mol Cancer (2019) 18(1):75. doi: 10.1186/S12943-019-0991-5 30940145 PMC6444571

[30] Chen H Wang L Zeng X Schwarz H Nanda HS Peng X. Exosomes, a new star for targeted delivery. Front Cell Dev Biol (2021) 9:751079. doi: 10.3389/FCELL.2021.751079 34692704 PMC8531489

[31] Fu S Wang Y Xia X Zheng JC. Exosome engineering: Current progress in cargo loading and targeted delivery. NanoImpact (2020) 20:100261. doi: 10.1016/J.IMPACT.2020.100261

[32] Gomzikova MO James V Rizvanov AA. Mitochondria donation by mesenchymal stem cells: current understanding and mitochondria transplantation strategies. Front Cell Dev Biol (2021) 9:653322. doi: 10.3389/FCELL.2021.653322 33898449 PMC8058353

[33] Zhang J Uchiyama J Imami K Ishihama Y Kageyama R Kobayashi T. Novel roles of small extracellular vesicles in regulating the quiescence and proliferation of neural stem cells. Front Cell Dev Biol (2021) 9:762293. doi: 10.3389/FCELL.2021.762293 34805169 PMC8601375

[34] Miyazaki K Wada Y Okuno K Murano T Morine Y Ikemoto T. An exosome-based liquid biopsy signature for pre-operative identification of lymph node metastasis in patients with pathological high-risk T1 colorectal cancer. Mol Cancer (2023) 22(1):2. doi: 10.1186/S12943-022-01685-8 36609320 PMC9817247

[35] Jang YO Ahn HS Dao TNT Hong JY Shin W Lim YM. Magnetic transferrin nanoparticles (MTNs) assay as a novel isolation approach for exosomal biomarkers in neurological diseases. Biomater Res (2023) 27(1):12. doi: 10.1186/S40824-023-00353-2 36797805 PMC9936675

[36] Liu C Su C. Design strategies and application progress of therapeutic exosomes. Theranostics (2019) 9:1015–28. doi: 10.7150/THNO.30853 PMC6401399 30867813

[37] Ghaffari K Moradi-Hasanabad A Sobhani-Nasab A Javaheri J Ghasemi A. Application of cell-derived exosomes in the hematological Malignancies therapy. Front Pharmacol (2023) 14:1263834. doi: 10.3389/FPHAR.2023.1263834 37745073 PMC10515215

[38] Sun W Xing C Zhao L Zhao P Yang G Yuan L. Ultrasound assisted exosomal delivery of tissue responsive mRNA for enhanced efficacy and minimized off-target effects. Mol Ther Nucleic Acids (2020) 20:558. doi: 10.1016/J.OMTN.2020.03.016 32334416 PMC7182664

[39] Bashyal S Thapa C Lee S. Recent progresses in exosome-based systems for targeted drug delivery to the brain. J Controlled Release (2022) 348:723–44. doi: 10.1016/J.JCONREL.2022.06.011 35718214

[40] Ke W Afonin KA. Exosomes as natural delivery carriers for programmable therapeutic nucleic acid nanoparticles (NANPs). Adv Drug Delivery Rev (2021) 176:113835. doi: 10.1016/J.ADDR.2021.113835 PMC8440450 34144087

[41] Marbán E. The secret life of exosomes: what bees can teach us about next-generation therapeutics. J Am Coll Cardiol (2018) 71:193–200. doi: 10.1016/J.JACC.2017.11.013 29325643 PMC5769161

[42] Jiang Y Zhang P Zhang X Lv L Zhou Y. Advances in mesenchymal stem cell transplantation for the treatment of osteoporosis. Cell Prolif (2021) 54(1):e12956. doi: 10.1111/CPR.12956 33210341 PMC7791182

[43] Stockwell J Abdi N Lu X Maheshwari O Taghibiglou C. Novel central nervous system drug delivery systems. Chem Biol Drug Des (2014) 83:507–20. doi: 10.1111/CBDD.12268 24325540

[44] Nair S Salomon C. Extracellular vesicles and their immunomodulatory functions in pregnancy. Semin Immunopathol (2018) 40:425–37. doi: 10.1007/S00281-018-0680-2 29616307

[45] Zheng Y Wang X Pan YJ Shi XF Yang L Lou YL. Orientin suppresses osteoclastogenesis and ameliorates ovariectomy-induced osteoporosis via suppressing ROS production. Food Sci Nutr (2023) 11:5582–95. doi: 10.1002/FSN3.3516 PMC10494641 37701239

[46] Li H Wang C Yao J Jin Y Song X Meng Q. Circ_0114581 promotes osteogenic differentiation of BMSCs via the MiR-155-5p/HNRNPA3 axis. Life Sci (2023), 122127. doi: 10.1016/J.LFS.2023.122127 37769807

[47] He X Wang Y Liu Z Weng Y Chen S Pan Q. Osteoporosis treatment using stem cell-derived exosomes: a systematic review and meta-analysis of preclinical studies. Stem Cell Res Ther (2023) 14(1):72. doi: 10.1186/S13287-023-03317-4 37038180 PMC10088147

[48] Sadat-Ali M Al-Dakheel DA Al-Turki HA Acharya S. Efficacy of autologous bone marrow derived Mesenchymal stem cells (MSCs), osteoblasts and osteoblasts derived exosome in the reversal of ovariectomy (OVX) induced osteoporosis in rabbit model. Am J Transl Res (2021) 13:6175. 34306356 PMC8290779

[49] Yahao G Xinjia W. The role and mechanism of exosomes from umbilical cord mesenchymal stem cells in inducing osteogenesis and preventing osteoporosis. Cell Transplant (2021) 30:9636897211057465. doi: 10.1177/09636897211057465 34814742 PMC8647230

[50] Li Z Hassan MQ Volinia S Van Wijnen AJ Stein JL Croce CM. A microRNA signature for a BMP2-induced osteoblast lineage commitment program. Proc Natl Acad Sci U S A (2008) 105:13906–11. doi: 10.1073/PNAS.0804438105 PMC2544552 18784367

[51] Gao Y Xiao F Wang C Wang C Cui P Zhang X. Long noncoding RNA MALAT1 promotes osterix expression to regulate osteogenic differentiation by targeting miRNA-143 in human bone marrow-derived mesenchymal stem cells. J Cell Biochem (2018) 119:6986–96. doi: 10.1002/JCB.26907 29741283

[52] Chen C Wang D Moshaverinia A Liu D Kou X Yu W. Mesenchymal stem cell transplantation in tight-skin mice identifies miR-151-5p as a therapeutic target for systemic sclerosis. Cell Res (2017) 27:559–77. doi: 10.1038/CR.2017.11 PMC5385608 28106077

[53] Li D Liu J Guo B Liang C Dang L Lu C. Osteoclast-derived exosomal miR-214-3p inhibits osteoblastic bone formation. Nat Commun (2016) 7:10872. doi: 10.1038/NCOMMS10872 26947250 PMC4786676

[54] Kalra H Adda CG Liem M Ang CS Mechler A Simpson RJ. Comparative proteomics evaluation of plasma exosome isolation techniques and assessment of the stability of exosomes in normal human blood plasma. Proteomics (2013) 13:3354–64. doi: 10.1002/PMIC.201300282 24115447

[55] Xiao F Zuo B Tao B Wang C Li Y Peng J. Exosomes derived from cyclic mechanical stretch-exposed bone marrow mesenchymal stem cells inhibit RANKL-induced osteoclastogenesis through the NF-κB signaling pathway. Ann Transl Med (2021) 9:798–8. doi: 10.21037/ATM-21-1838 PMC8246225 34268411

[56] Bolzoni M Toscani D Storti P Marchica V Costa F Giuliani N. Possible targets to treat myeloma-related osteoclastogenesis. Expert Rev Hematol (2018) 11:325–36. doi: 10.1080/17474086.2018.1447921 29495905

[57] Raimondo S Saieva L Vicario E Pucci M Toscani D Manno M. Multiple myeloma-derived exosomes are enriched of amphiregulin (AREG) and activate the epidermal growth factor pathway in the bone microenvironment leading to osteoclastogenesis. J Hematol Oncol (2019) 12(1):2. doi: 10.1186/S13045-018-0689-Y 30621731 PMC6325886

[58] Xu Z Liu X Wang H Li J Dai L Li J. Lung adenocarcinoma cell-derived exosomal miR-21 facilitates osteoclastogenesis. Gene (2018) 666:116–22. doi: 10.1016/J.GENE.2018.05.008 29730429

[59] Liu Z Zhang N Xin B Shi Y Liang Z Wan Y. Exosomes from LSD1 knockdown breast cancer cells activate osteoclastogenesis and inhibit osteoblastogenesis. Int J Biol Macromol (2023) 235:123792. doi: 10.1016/J.IJBIOMAC.2023.123792 36828097

[60] Yang Y Yuan L Cao H Guo J Zhou X Zeng Z. Application and molecular mechanisms of extracellular vesicles derived from mesenchymal stem cells in osteoporosis. Curr Issues Mol Biol (2022) 44:6346–67. doi: 10.3390/CIMB44120433 PMC9776574 36547094

[61] Liu S Liu W Yang Q Yang S Yang Y Fan L. Non-coding-RNA-activated core/chitosan shell nanounits coated with polyetheretherketone for promoting bone regeneration and osseointegration via osteoimmunology. ACS Appl Mater Interfaces (2023) 15:12653–68. doi: 10.1021/ACSAMI.2C19186 36868875

[62] Fan L Guan P Xiao C Wen H Wang Q Liu C. Exosome-functionalized polyetheretherketone-based implant with immunomodulatory property for enhancing osseointegration. Bioact Mater (2021) 6:2754–66. doi: 10.1016/J.BIOACTMAT.2021.02.005 PMC7897935 33665507

[63] Omidvar MH Soltani-Zangbar MS Zamani M Motavalli R Jafarpoor M Dolati S. The effect of osteoporotic and non-osteoporotic individuals’ T cell-derived exosomes on osteoblast cells’ bone remodeling related genes expression and alkaline phosphatase activity. BMC Res Notes (2022) 15(1):272. doi: 10.1186/S13104-022-06139-4 35941659 PMC9358836

[64] Ren Y Bäcker H Müller M Kienzle A. The role of myeloid derived suppressor cells in musculoskeletal disorders. Front Immunol (2023) 14:1139683. doi: 10.3389/FIMMU.2023.1139683 36936946 PMC10020351

[65] Leñero C Kaplan LD Best TM Kouroupis D. CD146+ Endometrial-derived mesenchymal stem/stromal cell subpopulation possesses exosomal secretomes with strong immunomodulatory miRNA attributes. Cells (2022) 11(24):4002. doi: 10.3390/CELLS11244002 36552765 PMC9777070

[66] Kouroupis D Kaplan LD Best TM. Human infrapatellar fat pad mesenchymal stem cells show immunomodulatory exosomal signatures. Sci Rep (2022) 12(1):3609. doi: 10.1038/S41598-022-07569-7 35246587 PMC8897449

[67] Kushioka J Chow SKH Toya M Tsubosaka M Shen H Gao Q. Bone regeneration in inflammation with aging and cell-based immunomodulatory therapy. Inflammation Regener (2023) 43(1):29. doi: 10.1186/S41232-023-00279-1 PMC10210469 37231450

[68] Hu Y Wang Y Chen T Hao Z Cai L Li J. Exosome: function and application in inflammatory bone diseases. Oxid Med Cell Longev (2021) 2021:6324912. doi: 10.1155/2021/6324912 34504641 PMC8423581

[69] Zhang S Liu Y Zhang X Zhu D Qi X Cao X. Prostaglandin E2 hydrogel improves cutaneous wound healing via M2 macrophages polarization. Theranostics (2018) 8:5348. doi: 10.7150/THNO.27385 30555551 PMC6276096

[70] Zhang L Liu J Zhou C. Current aspects of small extracellular vesicles in pain process and relief. Biomater Res (2023) 27(1):78. doi: 10.1186/S40824-023-00417-3 37563666 PMC10416402

[71] Cao G Meng X Han X Li J. Exosomes derived from circRNA Rtn4-modified BMSCs attenuate TNF-α-induced cytotoxicity and apoptosis in murine MC3T3-E1 cells by sponging miR-146a. Biosci Rep (2020) 40(5):BSR20193436. doi: 10.1042/BSR20193436 32400849 PMC7251325

[72] Qiu M Zhai S Fu Q Liu D. Bone marrow mesenchymal stem cells-derived exosomal microRNA-150-3p promotes osteoblast proliferation and differentiation in osteoporosis. Hum Gene Ther (2021) 32:717–29. doi: 10.1089/HUM.2020.005 33107350

[73] Xie X Xiong Y Panayi AC Hu L Zhou W Xue H. Exosomes as a novel approach to reverse osteoporosis: A review of the literature. Front Bioeng Biotechnol (2020) 8:594247. doi: 10.3389/FBIOE.2020.594247 33195163 PMC7644826

[74] Sun W Zhao C Li Y Wang L Nie G Peng J. Osteoclast-derived microRNA-containing exosomes selectively inhibit osteoblast activity. Cell Discovery (2016) 2:16015. doi: 10.1038/CELLDISC.2016.15 27462462 PMC4886818

[75] Zhu X Badawi M Pomeroy S Sutaria DS Xie Z Baek A. Comprehensive toxicity and immunogenicity studies reveal minimal effects in mice following sustained dosing of extracellular vesicles derived from HEK293T cells. J Extracell Vesicles (2017) 6(1):1324730. doi: 10.1080/20013078.2017.1324730 28717420 PMC5505007

[76] Zhang Y Liu Q Zhang X Huang H Tang S Chai Y. Recent advances in exosome-mediated nucleic acid delivery for cancer therapy. J Nanobiotechnol (2022) 20(1):279. doi: 10.1186/S12951-022-01472-Z PMC9194774 35701788

[77] Gurunathan S Kang MH Jeyaraj M Qasim M Kim JH. Review of the isolation, characterization, biological function, and multifarious therapeutic approaches of exosomes. Cells (2019) 8(4):307. doi: 10.3390/CELLS8040307 30987213 PMC6523673

[78] Aguiar Koga BA Fernandes LA Fratini P Sogayar MC Carreira ACO. Role of MSC-derived small extracellular vesicles in tissue repair and regeneration. Front Cell Dev Biol (2023) 10:1047094. doi: 10.3389/FCELL.2022.1047094 36935901 PMC10014555

[79] Zhang E Phan P Zhao Z. Cellular nanovesicles for therapeutic immunomodulation: A perspective on engineering strategies and new advances. Acta Pharm Sin B (2023) 13:1789–827. doi: 10.1016/J.APSB.2022.08.020 PMC10213819 37250173

